# Aging research from bench to bedside and beyond: What we learned from Sammy Basso

**DOI:** 10.1111/acel.14414

**Published:** 2024-12-11

**Authors:** Giovanna Lattanzi, Chiara Lanzuolo, Eleonora Cugudda, Lorenzo Maggi, Luisa Politano, Olaya Santiago‐Fernández, Giulia Ricci, Stefano Squarzoni, Carlos Lopez‐Otin, the Italian Network for Laminopathies

**Affiliations:** ^1^ CNR Institute of Molecular Genetics “Luigi Luca Cavalli‐Sforza” Unit of Bologna Bologna Italy; ^2^ IRCCS Istituto Ortopedico Rizzoli Bologna Italy; ^3^ ITB‐CNR, Institute of Biomedical Technologies, National Research Council Segrate Italy; ^4^ INGM Istituto Nazionale Genetica Molecolare “Romeo Ed Enrica Invernizzi” Milan Italy; ^5^ AIDMED, Associazione Italiana Distrofia Muscolare di Emery‐Dreifuss OdV Modena Italy; ^6^ Neuroimmunology and Neuromuscular Diseases Fondazione IRCCS Istituto Neurologico Carlo Besta Milan Italy; ^7^ Cardiomyology and Medical Genetics Unit Luigi Vanvitelli Campania University Naples Italy; ^8^ Department of Developmental and Molecular Biology Albert Einstein College of Medicine Bronx New York USA; ^9^ Department of Clinical and Experimental Medicine University of Pisa Pisa Italy; ^10^ Centre de Recherche Des Cordeliers, Inserm U1138, Université Paris Cité Sorbonne Université Paris France

Sammy Basso, a passionate advocate and researcher in the field of premature aging, passed away on 6 October 2024, at the age of 28. Sammy was affected by Hutchinson‐Gilford Progeria (HGPS), one of the most severe and life‐threatening accelerated aging diseases, affecting children shortly after birth (Cenni et al., 2020; Gordon et al., 2014). Yet, Sammy instead of succumbing to his condition, embraced it with remarkable resilience and a determination to make a difference. He made positive thinking his philosophy of life, and his infectious spirit left all of us astonished and in disbelief when he ultimately passed away. Researchers involved in laminopathy and aging research have lost not only a colleague, a source of inspiration, enthusiasm, and positivity, but also a dear friend.

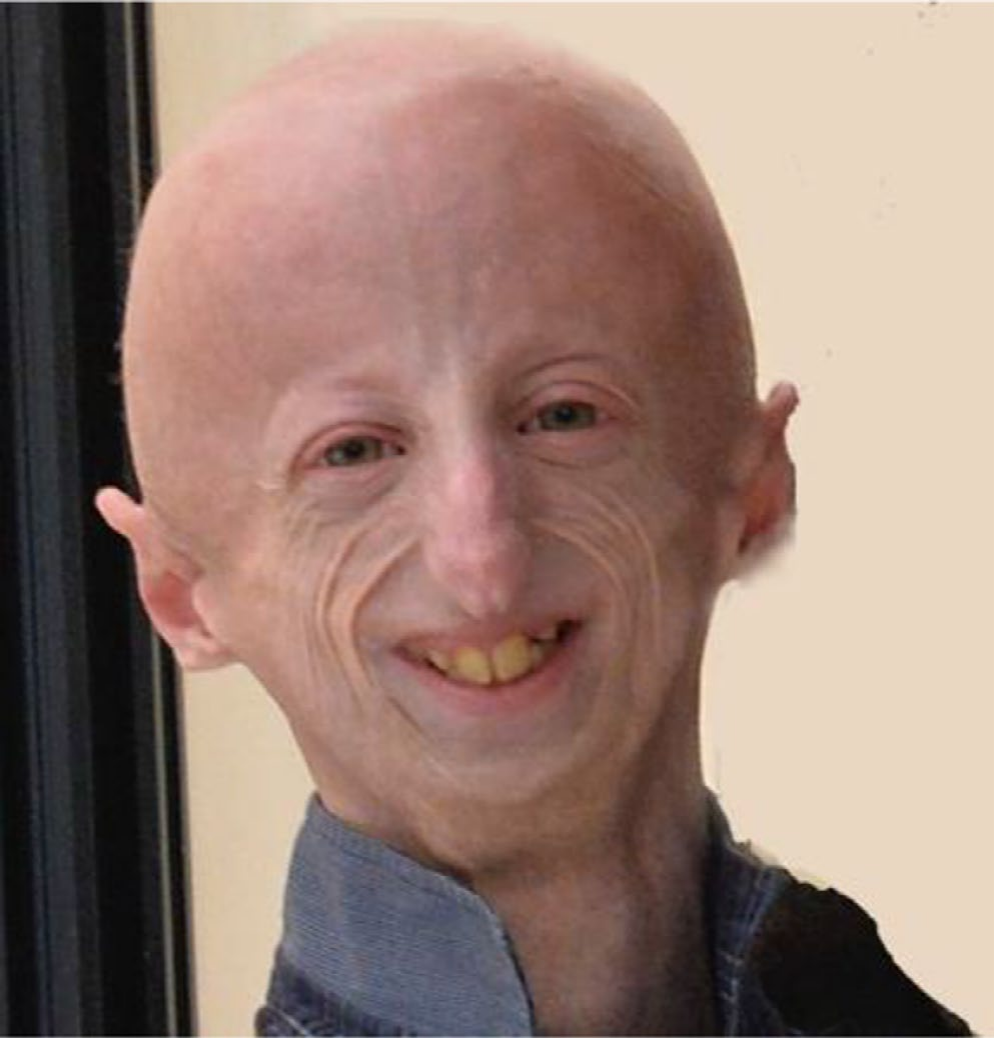

Since his teenage years, Sammy demonstrated an extraordinary awareness of his condition, far surpassing the self‐awareness of many adults. With his innate positivity, he led a fulfilling life, achieving numerous goals, inspiring both patients and researchers alike. We witnessed him discuss progeria with the same rigor as any other researcher, we saw him working to understand molecular mechanisms in experimental models and clinical presentations in patients, with the final aim to find a cure. Even when discussing the prospect of a cure that hardly could have been found in a few years, he remained focused on younger patients, often stating: “I'm working for them”.

In 2017, he wrote a message intended to be read at his funeral, expressing his thoughts on living with progeria and his positive outlook on life: “*I don't know when or how I will leave this world, and many may say that I have lost my battle against the disease. Don't listen to them! There was never a battle to fight; there was only a life to embrace as it is, challenging yet beautiful and extraordinary. There is no reward, no punishment, just a gift by God*” and “*If you'd like to remember me, don't waste too much time on various rituals, pray, of course, but also take some glasses, toast to my health and yours, and embrace joy. I've always enjoyed being around others, and that's how I'd like to be remembered*”.

In the last decade, Sammy emerged as one of the most effective science communicators among young people and non‐expert audiences worldwide. Together with the American Progeria Research Foundation (https://www.progeriaresearch.org/) he brought tremendous mediatic attention to this rare disease, facilitating the diagnosis of several children worldwide. His speeches raised interest in progeroid rare diseases and fostered a positive attitude towards the challenges living with progeria presents in life and life itself.

One of Sammy's dreams materialized with the establishment of the Italian Network for Laminopathies (NIL) in 2009 (https://www.igm.cnr.it/laminopatie/en/). At the age of 14 years, he was among the founders of NIL, and since then, Sammy and his association, the AIProSaB (https://www.aiprosab.org/), have encouraged all NIL partners to become increasingly engaged, providing motivation and support in various aspects, from data sharing to project drafting, from meeting organization to website development, his last impressive contribution.

As a researcher, Sammy was deeply committed to understanding the complexities of progeria. He was curious, consistently seeking new perspectives, both within and outside of mainstream science. His engagement in gene therapy for HGPS spanned from the preparation of his graduation thesis at Oviedo University in Spain until his passing. As a member of the Progeria Research Foundation, he collaborated closely with researchers trying to obtain FDA approval for the first gene therapy for progeria. A few years ago, he was engaged in obtaining EMA approval of lonafarnib, now available as at‐home oral therapy for Progeria patients. He was recently engaged in the working group for the optimization of surgical interventions to relieve critical aortic stenosis in HGPS patients (Gordon et al., 2024). Sammy directly contributed with both his brilliant mind and his own hands to the development of a CRISPR/Cas9‐based therapy for HGPS which was successfully tested in a murine model of progeria (Osorio et al., 2011; Santiago‐Fernández et al., 2019). His research at the CNR Institute of Molecular Genetics in Bologna also included studying interleukin 6‐dependent mechanisms in HGPS (Squarzoni et al., 2021) and exploring potential immunological therapies, a focus that began with his Master's thesis in Molecular Biology, which he completed with honors at the University of Padua; as well as work on fat loss in HGPS, a study which is currently in submission.

In all these endeavors, his optimism, cheerfulness, humor, and kindness illuminated the lives of all who had the privilege of knowing him.

He leaves behind a rich legacy of research, compassion, and inspiration. Perhaps, the most significant lesson we learned from Sammy, is to have a fresh and open perspective on science, characterized by a shared curiosity and enthusiasm, a positive view of new findings, realism, and a determination to overcome obstacles with rigorous and open‐minded approaches.

We honor Sammy's memory with deep gratitude for the inspiration he provided and the impact he made in the world of science and beyond.

## AUTHOR CONTRIBUTIONS

All authors contributed equally to this article.

## CONFLICT OF INTEREST STATEMENT

No conflicts of interest.

## Data Availability

Data sharing not applicable to this article as no datasets were generated or analysed during the current study.
